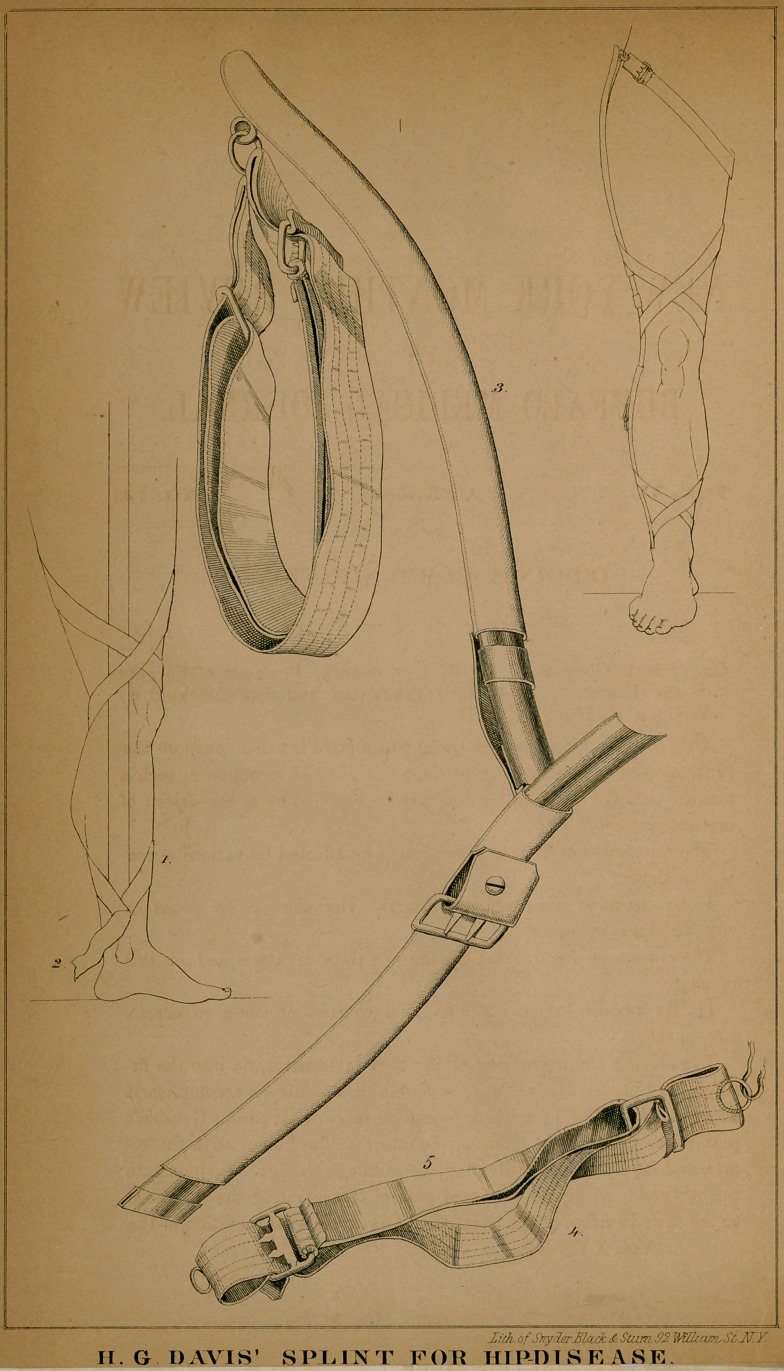# On the Mechanical Means Adopted in the Treatment of Morbus Coxarius

**Published:** 1860-04

**Authors:** H. G. Davis

**Affiliations:** 67 Union Place


*On the Mechanical Means adopted in the Treatment of Morbus Coxarius.* By H. G. Davis, M.D. (*With a Plate.*)

The results obtained from the present modes of treating morbus coxarius are not so satisfactory as to require an apology when a new method is proposed, especially when that method, in some important particulars, has thus far in its application proved perfectly satisfactory.

It is my intention to confine the contents of this paper almost exclusively to a description of a method of treating this disease which I have pursued for twelve years; and as it has never been brought before the profession, it becomes necessary to describe it minutely. As I proceed in the description of the apparatus used, and the manner of its application, it will be somewhat necessary that I indicate the reasoning which guided me towards its adoption.

The several parts employed in the treatment are four strips of adhesive plaster, (see Plate, Fig. 1;) a roller to confine them to the limb, firm webbing to be attached to the lower end of the adhesive plasters when upon the limb, (Fig. 2;) a cord, pulley, and weight, for extension when upon a bed or couch; a corrugated steel splint, (Fig. 3,) with a perineal band, composed of two parts—an inelastic, (Fig. 4,) and an elastic, (Fig. 5)—arranged in a peculiar manner, so as to keep up extension while the patient may take exercise within the house or in the open air.

When called to treat a case of morbus coxarius, I bring the tibia in a line with the femur, but attempt no change in the direction of the latter if it is not parellel with the body. If the femur is flexed upon the pelvis, the body should be raised until the limb will lie extended upon the mattress or couch. Adhesive straps are placed upon each side of the limb, in the following manner: First, double over one inch of each adhesive strap designed for the sides of the limb, bringing the adhesive surfaces in contact for the purpose of increasing the strength of the part, to which a firm inelastic webbing is to be attached. The strip for the outside of the limb commences with the folded end mentioned, at a point one inch above the external malleolus, and extends to the region of the great trochanter; that upon the inside, from one inch above the internal malleolus to within one inch of the pubis. Upon the lower end of the adhesive strap, on the outside of the limb, I commence with a narrower and longer one, and run it spirally around the limb until it reaches the upper end of the longitudinal straps; another starts from the same point upon the outside of the limb, but winds in the opposite direction. These spiral strips accomplish a two-fold purpose: they connect the outside longitudinal adhesive plaster with that upon the inside of the limb, so that any extension made upon the outside plaster is shared by that upon the inside. This arrangement also secures the bottom of the outside longitudinal plaster from being displaced laterally when the splint is applied, always retaining it in a line with the limb. The width of these plasters varies from an inch and a quarter to two inches and a half, according to the size of the patient.

When the extension is made by the splint, it is always upon the outside of the limb; at other times it can be made from both sides; the latter mode of extension supports the sides of the foot equally, and is therefore preferable when the patient is upon the bed or couch.

Before proceeding further, I would say a word upon the character of the adhesive plaster to be used for such purposes, as that ordinarily used for dressing wounds will only disappoint the surgeon. It should be spread upon twilled goods, as they are more elastic; and when the extension is made the parts first affected will yield until the whole surface of the plaster bears a portion of the draught; whereas plaster upon plain cloth draws only in a straight line, therefore is only applicable to an even surface.

The material of the plaster should not only be good, but it should have been spread upon the cloth for at least one year, and it is still better if two years old; age oxydizes the oil, rendering it resinous, so that the oily secretions from the skin do not readily soften it. Plaster of this description I have had remain upon an adult for seven months, sustaining a weight of twelve pounds every night, and not unfrequently during the day a considerable portion of the entire weight of the body.

After the application of the adhesive straps in the manner described, the limb should be covered with a bandage from the foot to the pelvis, to secure firm adhesion of the plasters, also to prevent their edges from being raised by coming in contact with the clothing, or by the hands of the patient during sleep; this bandage should be affixed to the limb for some hours, with the patient warm in bed, before any draught is made upon the adhesive straps, that they may become firmly adherent to the limb; after this time has elapsed, a weight, varying from two to six pounds, according to the strength of muscle and the sensibility of the joint, may be attached to the adhesive straps upon each side of the limb by a cord that runs over a small pulley secured to the foot of the bed or couch; the top of the pulley being a little above a line with the centre of the limb. This weight should be increased from day to day, until a general sensation of fatigue is felt in the entire limb to an unpleasant extent, and then diminished until it just falls short of this point. The extension is first made at that angle with the body that I find the limb; as the tenderness of the joint subsides, the body should be gradually lowered until it is brought in a line with the limb; when this is effected, the splint can be applied, and the patient put upon crutches and permitted to exercise.

Upon the lower end of the adhesive straps attached to the outside of the limb, I secure a piece of firm, strong webbing, of sufficient width to support the weight of the patient; this passes over the end of the splint, and is inserted into a buckle on its outside, near the joint.

The counter-extension is now ready for the splint; this I make of steel, of sufficient width and thickness, that when corrugated as it is in the splint, it will sustain the weight of the patient without yielding; and it is surprising how little weight of material it requires to fulfill these indications when put into the corrugated form. A strip one inch and a half wide, of No. 16 rolled cast-steel, when corrugated, will sustain, endwise, the weight of an adult without yielding.

The splint has a joint near the knee, for the purpose of rendering it easy of application. The lower portion overlaps the upper, and when the splint is brought up to the limb, and the extension made, a piece of steel in the form of a band slides over the end of the overlapping portion, and secures the joint. The bottom of the splint, in some cases, I make of a separate piece, with holes for altering the length, and secured by screws to the other; but without this the splint admits of a difference in the length of the limb of two or three inches, which is frequently all the alteration that is necessary. If the webbing attached to the plaster is some wider than the bottom of the splint, it will contract over the corners sufficiently to retain the splint in its fold; to make it still more secure, the lower end of the splint can be cut into, forming slight grooves, into which the threads of the webbing will draw, thus preventing displacement. Just below the joint, in the splint, is the button for the attachment of the buckle, before mentioned. Quite at the top, and inside of the splint, is an eye, through which runs the catgut attached to the two ends of a perineal or extending band, and forms a part of it when the whole is applied to the patient; the catgut, passing through this eye unconfined, allows the upper portion of the splint to traverse back and forth without disturbing the perineal band. All of that portion of the splint that passes above the hip-joint has a motion, of which the joint is the centre. By this arrangement, allowing the catgut to traverse in the eye of the splint, the perineal band is not disturbed by any motions of the limb, and all irritation from motion is avoided. The whole of the splint is covered with leather, with the exception of a small space at the bottom, and that occupied by the joint and its fastenings; between this and the limb is a flat piece of leather, to prevent irritation from this part. The perineal or extending band is a very simple, though a very important contrivance. It is so arranged that any amount of extension can be made; yet, when the patient steps upon the limb, it yields no further.

There is, first, an elastic band made of rubber, or rubber webbing, to which two buckles are attached, one at each end. In addition, there is a piece of inelastic webbing, that is from eight to twelve inches longer than the elastic band mentioned; this passes through the buckles in such a direction that when draught is made upon each end, the buckles take hold of the webbing and confine it; by this arrangement of the two bands they can be buckled at different lengths; by leaving the outside inelastic band loose between the buckles, any draught applied at the ends of the inelastic will be communicated to the elastic band, extending it until it is of the same length as that portion of the inelastic band contained between the buckles; then the latter will prevent any further extension. By this arrangement it will be perceived how a certain amount of extension can be kept up; yet when any additional labor is thrown upon the splint, as when the patient leans upon the limb, it will yield no further. The loose ends of the inelastic webbing I fold back upon themselves, and introduce into the buckles; where the folds come, a steel ring is stitched on securely, for the purpose of fastening the piece of catgut that is to pass through the eye at the upper end of the splint; the end of the inelastic webbing is folded back, as mentioned, that the length of the perineal band may be varied without untying the catgut. Both ends of the perineal band are secured to the catgut after the latter is placed in the eye at the top of the splint; the whole forming a loop that can be put upon the limb over the foot without disturbing any part. For a cushion beneath the perineal band, I use old table linen, or old napkins that are soft; they should be folded an inch wider than the perineal band, and a little longer; after folding, the parts should be caught with a needle and thread, to prevent their displacement.

These cushions should be changed every second day, particularly during warm weather, as they collect the secretions, and then are liable to excoriate the parts. For the first few days, care should be taken that the splint is not kept on, after anything like a sensation of heat or smarting is experienced in the groin. If proper care be observed at first, the parts beneath the perineal or extension band will have become so hardened as to render any excoriation improbable.

Whenever the splint is removed, the extension by means of the weight should be applied, that the diseased surfaces of the joint may be constantly separated. The splint is intended to fit closely to the outside of the limb, and to be worn inside the stocking; that this may be done, and also that it might be worn unperceived, I have avoided all additions to its thickness. Irregularities upon its surface have been guarded against, as they would soon wear a hole through the clothing, and thus become obnoxious. The eye for the catgut is the most simple form of accomplishing the object, is never liable to get out of order, and in my hands has not originated an objection. If the mechanic who constructs it is not aware of its use, he may leave it so unfinished as to fray the catgut, but this is no fault of the principle. I have an adult patient that has used the same piece of catgut for ten months.

This plan of treatment has been foreshadowed in the various contrivances and operations adopted by surgeons, and with varying success in proportion as they carried out the principle involved, and, as I conceive, fully applied in the mode just described.

It is not an unfrequent occurrence that a failure results from an imperfect application of a correct principle. Hence, we often see the revival of some principle of treatment, followed by success, that has previously been condemned, because of its imperfect application.

I have delayed bringing the subject of this paper before the profession until time had given me an opportunity, not only to overcome any minor difficulties that might arise, but to test its application, and compare the results with the modes heretofore practiced. It is an unfortunate circumstance that so many new things are hurried before the profession in a crude state, to be condemned or die of neglect, when they would have been highly useful if the inventor or discoverer had taken time to digest and mature his plans, and then apply them until all objections or difficulties in their use should be overcome.

The advantages of this plan of treatment are:

1st. It relieves all suffering after the limb is brought down in a line with the axis of the body, and the sensibility of the joint has had time to subside; but from the commencement, there is a decided mitigation. The time requisite to bring the limb down varies from one to twenty days, according to the tenderness of the joint and the contraction of the muscles. I give it as a rule to the attendants, that whenever the patient complains of pain, they may be certain there is not the proper amount of extension upon the limb.

2d. It retains the limb in the best position as to length, &c., whatever may be the result to the head of the bone.

3d. It puts the diseased parts in the best position for their restoration with a perfect joint, as it relieves the pressure upon the head of the bone, while at the same time, it admits of motion, which increases the recuperative energy of the parts, inasmuch as it increases their vitality.

4th. In consequence of the favorable results mentioned, the patient's life will rarely if ever be sacrificed to the disease.

The relief afforded the parents and friends of the patient might be mentioned, as there are few diseases that make a greater demand upon the sympathies and physical efforts of parents and friends than this.

In a former paper, I incidentally mentioned the relief from irritation afforded by separating the head of the bone from the upper portion of the acetabulum. I have had patients with very depraved constitutions, where large abscesses had formed about the joint, in whom hectic fever would have been sure to follow their opening under the old mode; but while extension was kept up, not an unpleasant symptom followed, not even so much as any impairment of the appetite. This favorable result I have seen follow the opening of abscesses both in Pott's and hip disease, when the parts were kept separated.

It will not be expected that every case of morbus coxarius, although treated upon this plan from the commencement of the disease, will recover with a perfect joint; yet we can safely rely upon a far larger per centage than by any other mode; in addition, whatever may be the point at which the disease stops, the limb will be left in the best condition possible, considering the loss of structure sustained. If the head of the femur should be entirely destroyed, the limb should be kept at its full length; and if anchylosis takes place, it does so without material shortening of the limb. If a patient should recover from the disease while wearing the apparatus, great care is requisite that it be not laid aside too soon; the necessity of this caution becomes evident, when we consider upon how small a surface of the head of the bone, or of the cavity of the acetabulum, must rest the whole weight of the body, and frequently many times its weight, as in jumping, &c.

Again, this very small surface is the portion that has just recovered from disease, or, in other words, is a fresh cicatrix, the vitality of which is less than that of the original structure. There is yet another consideration: if inflammation takes place in this part, it will be of a far more active character than that with which it was at first affected, and will very probably speedily result in suppuration if not subdued. The remedial measures should be much more active, particularly the local treatment, than in an ordinary case of hip disease.

It will be observed that the treatment I have presented is entirely mechanical, therefore does not interfere with any constitutional or local medication, which any gentleman may consider advantageous. On this part of the treatment I do not design to speak at the present time, but reserve it for a future paper.

67 Union Place.

## Figures and Tables

**Figure f1:**